# Cervical Cancer Outcomes and Toxicity in HIV-Positive Women Treated With Radiation or Chemoradiation: A Systematic Review

**DOI:** 10.7759/cureus.99855

**Published:** 2025-12-22

**Authors:** Florence Mutua, Sheen Dube, Anuraag Shrivastav, Saranya Kakumanu, Vibhay Pareek

**Affiliations:** 1 Radiation Oncology, CancerCare Manitoba, Winnipeg, CAN; 2 Biochemistry, University of Winnipeg, Winnipeg, CAN; 3 Biology, University of Winnipeg, Winnipeg, CAN; 4 Paul Albrechtsen Research Institute, CancerCare Manitoba, Winnipeg, CAN

**Keywords:** cervical cancer, chemoradiation, hiv, radiotherapy, survival, toxicity

## Abstract

Cervical cancer disproportionately affects HIV-positive women, especially in low- and middle-income countries (LMICs), with a sixfold increased risk due to immunosuppression and persistent HPV infection. This systematic review evaluates the clinical outcomes and toxicities associated with radiotherapy or chemoradiation in HIV-positive compared to HIV-negative women with cervical cancer. Following the Preferred Reporting Items for Systematic Reviews and Meta-Analyses (PRISMA) guidelines, we conducted a comprehensive search on August 15, 2023 and an update on June 20, 2025, of Ovid Medline, EMBASE, and ClinicalTrials.gov from database inception to June 2025, as well as a manual review of citations. This process identified 18 cohort studies (11 prospective and seven retrospective), including 2,790 HIV-positive and 4,064 HIV-negative patients treated for cervical cancer with radiation-based treatments. The majority of studies (15 out of 18) were conducted in sub-Saharan Africa. The findings indicated that HIV-positive patients had a 33% lower overall survival (OS) compared to HIV-negative patients (1.33 (95% confidence interval (CI) 1.08-1.63), p = 0.0080). Women living with HIV had an increased risk of mortality, exhibiting a relative risk of 1.27 (95% CI 1.13-1.43, p = 0.0080). They also showed a trend toward lower complete response rates, with a relative risk of 0.92 (95% CI: 0.79-1.06, p = 0.1595). Treatment toxicity was reported in 10 studies, but the grading scales and toxicity reporting differed across studies. Some studies reported differences in toxicities by HIV status, while others found no difference. However, data regarding the impact of concurrent antiretroviral therapy (ART) were not available for assessment on their influence on treatment outcomes and toxicities. The findings underscore the significant research deficiencies in the management of cervical cancer among HIV-positive women undergoing radiation therapy. There is a pressing need for targeted research to elucidate clinical and toxicity outcomes, thereby refining management strategies for this specific patient cohort.

## Introduction and background

Cervical cancer, the fourth most common cancer among women globally, accounts for over 600,000 new cases and 300,000 deaths annually [1]. Low- and middle-income countries (LMICs), particularly in sub-Saharan Africa, bear a disproportionate burden due to limited healthcare access and high HIV prevalence [2]. HIV-positive women face a six-fold increased risk of cervical cancer due to immunosuppression, which exacerbates human papillomavirus (HPV) persistence and accelerates progression to invasive disease [3-5].

Radiotherapy, often combined with cisplatin-based chemotherapy, is the standard treatment for locally advanced cervical cancer [6]. However, HIV-positive patients experience unique challenges, including higher treatment-related toxicities, potential drug-drug interactions between antiretroviral therapy (ART) and cancer treatments, and adherence issues [7-10]. Conflicting data on survival and toxicity outcomes in HIV-positive patients highlight the need for evidence synthesis [11-13]. This systematic review evaluates clinical outcomes, toxicities, and prognostic factors in HIV-positive women undergoing radiotherapy or chemoradiation for cervical cancer, aiming to guide clinical practice and policy.

## Review

Methods

This review adhered to the Preferred Reporting Items for Systematic Reviews and Meta-Analyses (PRISMA) guidelines [14] and the Cochrane Handbook [15]. The protocol (see Appendix A) was developed in accordance with PRISMA-P standards [16]. The review utilized the PICO (Population, Intervention, Comparator, Outcome) framework: the population was patients with cervical cancer, the intervention group was HIV-positive patients, the comparator group was HIV-negative patients, and the outcomes assessed were survival data and toxicities. The aim was to compare the clinical outcomes and toxicities of cervical cancer patients treated with radiotherapy or chemoradiation based on their HIV status. 

Eligibility Criteria

Studies eligible for inclusion were retrospective or prospective cohort studies, as well as randomized controlled trials, that compared clinical outcomes and toxicities based on HIV status in adult women (aged 18 years or older) with early or locally advanced cervical cancer who were treated with radiotherapy or chemoradiation. Only studies published in English were included. Studies focusing on metastatic disease were excluded. Conference abstracts, reviews, animal studies, and editorials were also excluded.

Search Strategy

Comprehensive searches were conducted in Ovid Medline, EMBASE, and ClinicalTrials.gov (JL) on August 15, 2023, with no publication date limits applied. The searches were supplemented by hand-searching reference lists. Searches were updated on June 23, 2025. Search strategies are provided in Table 1.

**Table 1:** Search strategies

Ovid MEDLINE(R) and EPUB ahead of print, in-process, in-data-review & other non-indexed citations and daily <1946 to June 20, 2025>	
1	exp HIV/ or exp HIV Infections/ or HIV Seropositivity/ or HIV Seroprevalence/
2	people living with HIV.mp. or plwh*.ti,ab.
3	1 or 2
4	(cervical cancer* or cervical neoplasm*).mp. or Uterine Cervical Neoplasms/
5	(chemotherap* or cisplatin).mp.
6	exp Radiotherapy/
7	(radiotherap* or radiation therap*).mp.
8	exp Chemoradiotherapy/
9	(chemoradiat* or chemo radiat*).mp.
10	5 or 6 or 7 or 8 or 9
11	exp Drug Therapy/ and (6 or 7)
12	10 or 11
13	3 and 4 and 12
EMBASE <1974 to 2025 Week 25>	
1	human immunodeficiency virus infection/ or exp acquired immune deficiency syndrome/ or exp acute hiv infection/ or exp human immunodeficiency virus 1 infection/ or exp human immunodeficiency virus 2 infection/
2	exp Human immunodeficiency virus/
3	1 or 2
4	(cervical cancer* or cervical neoplasm*).mp. or exp uterine cervix cancer/ or exp uterine cervix carcinoma/
5	(chemotherap* or cisplatin).mp.
6	exp radiotherapy/
7	exp chemoradiotherapy/
8	exp cancer chemotherapy/
9	exp cancer radiotherapy/
10	cancer therapy/
11	(5 or 8) and (6 or 9)
12	7 or 10 or 11
13	3 and 4 and 12
14	5 or 6 or 7 or 8 or 9 or 10
15	3 and 4 and 14

Study Selection Process

The identified studies were imported into Covidence for screening [17]. Two reviewers (VP and SD) independently screened the study titles and abstracts using the eligibility criteria, followed by a full-text assessment of those identified as potentially eligible. Any discrepancies were resolved through discussion and consensus.

Data Extraction

A data extraction form was developed in Covidence. Two authors (FM, VP) independently extracted data, including study characteristics, patient demographics, cancer stage, treatment details, outcomes, toxicities, and HIV-related data (CD4 counts, viral load, ART regimens). A third author resolved any discrepancies.

Risk-of-Bias Assessment

Each included study was evaluated for risk of bias using the Newcastle-Ottawa quality assessment for cohort studies [18]. This tool assesses cohort selection, comparability, and the assessment of outcomes and follow-up periods. The influence of studies that were assessed as of poor quality was assessed in a sensitivity analysis.

Data Synthesis and Analysis

Data were exported to Microsoft Excel (Microsoft Corp., Redmond, WA, USA) and analyzed using R version 4.4.1 (R Foundation for Statistical Computing, Vienna, Austria, https://www.R-project.org/) and RevMan version 5.4 (Review Manager, The Cochrane Collaboration, London, UK) [19]. Forest plots were used to display effect estimates for outcomes by HIV status using random-effects models to account for heterogeneity [20,21]. Statistical heterogeneity was assessed using the I² statistic, with values below 40% considered unimportant heterogeneity and those above 75% indicating considerable heterogeneity. Where significant heterogeneity was identified, subgroup analysis was conducted by study design, location, and quality. A sensitivity analysis was conducted to assess the robustness of the pooled effect estimate. Statistics included risk ratios (RR), hazard ratios (HR), confidence intervals, and p-values. The association between HIV and overall survival (OS) was assessed by pooling HRs using the random-effects inverse-variance method in RevMan, with a random-effects meta-analysis to account for heterogeneity across studies. The data entered were the HRs with the 95% confidence intervals or the p-values.

Results

Study Selection Process and Quality Assessment

**Figure 1:**
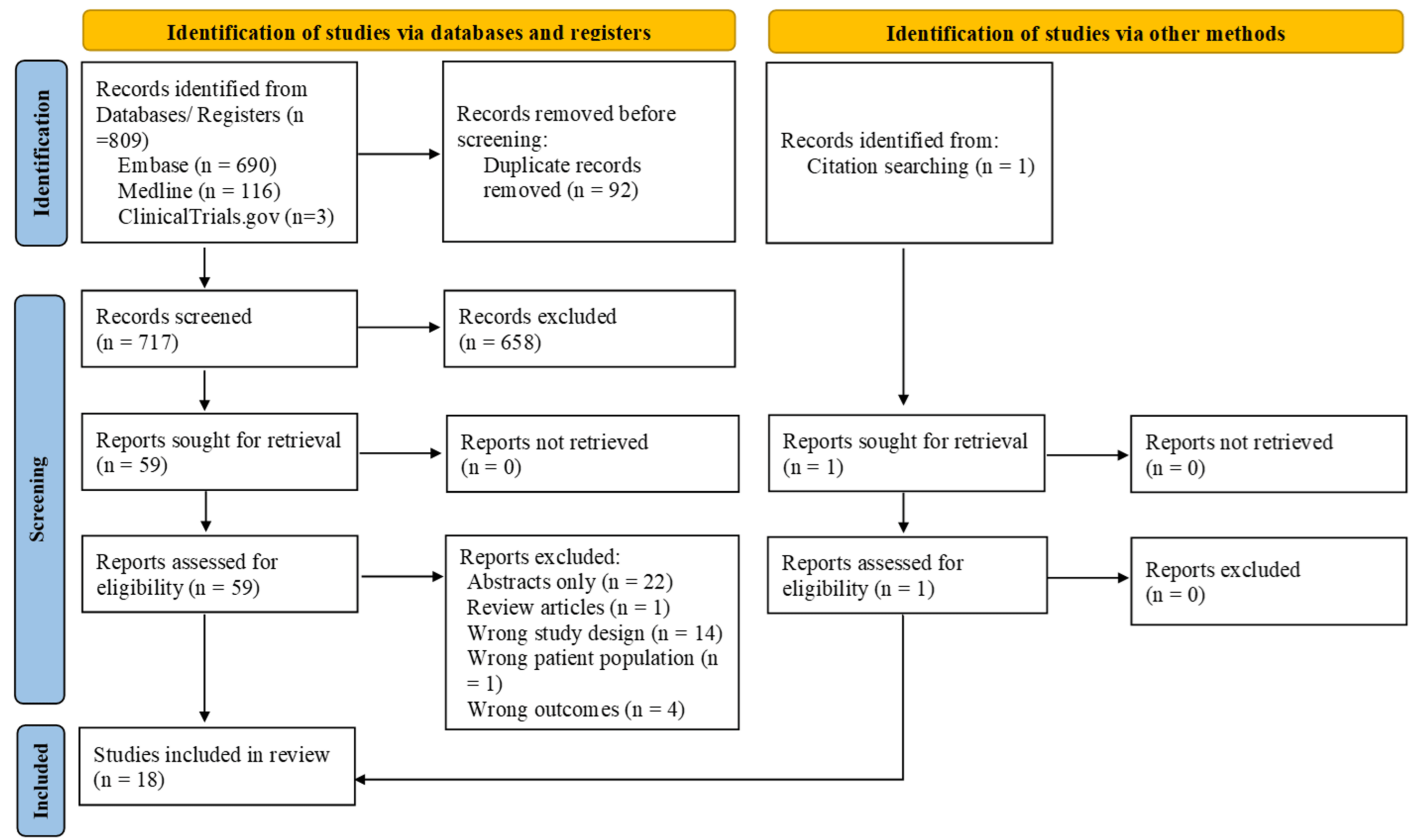
Preferred Reporting Items for Systematic Reviews and Meta-Analyses (PRISMA) flowchart illustrating the study selection process

The study selection process is illustrated in Figure 1, showing the steps taken at each stage and the reasons for excluding studies in accordance with the PRISMA 2020 guidelines. A total of 809 studies were identified through the search conducted in Ovid Medline, EMBASE, and ClinicalTrials.gov, as well as one additional study identified through a search of citations. After removal of 92 duplicates in Covidence, two authors (VP and SD) independently screened the 717 titles and abstracts, excluding those irrelevant to the study. Reasons for exclusion included the unavailability of full texts, or an incorrect study design, study population, or outcomes.

The quality of the included studies was assessed using the Newcastle-Ottawa Quality Assessment form [18]. Out of the 18 studies, 16 were rated as having good quality, while the remaining two were rated as poor quality because they received a zero score in the comparability domain. These two studies did not indicate whether they controlled for confounding factors (Table 2) [22,23].

**Table 2:** Quality assessment of the included studies using the Newcastle-Ottawa tool The studies were scored with a maximum of one star (*) for each item in the Selection and Outcome categories and a maximum of two stars (**) in the Comparability category [18].

Study	Selection				Comparability (two stars)	Outcome			Quality
	HIV+ cohort (one star)	HIV- cohort (one star)	Ascertainment of exposure (one star)	Outcome of interest not present at start of study (one star)		Assessment of outcome (one star)	Length of follow-up (one star)	Adequacy of follow-up (one star)	
Dryden-Peterson 2016 [24]	★	★	★	★	★★	★	★	★	Good
Ferreira 2017 [25]	★	★	★	★	★★	★	★	★	Good
Gichangi 2006 [26]	★	★	★	★	★★	★	★	★	Good
Grover 2018 [27]	★	★	★	★	★	★	★		Good
Grover 2021 [28]	★	★	★	★	★★	★	★		Good
Grover 2022 [29]	★	★	★	★	★★	★	★		Good
Grover 2025 [30]	★	★	★	★	★★	★	★	★	Good
Kavuma 2022 [22]	★	★	★	★		★	★		Poor
Kigula-Mugambe 2006 [23]	★	★	★	★		★	★		Poor
MacDuffie 2021 [31]	★	★	★	★	★★	★	★		Good
Mangena 2015 [32]	★	★	★	★	★	★	★		Good
Meghani 2024 [33]	★	★	★	★	★★	★	★		Good
Simonds 2012 [34]	★	★	★	★	★★	★	★	★	Good
Simonds 2015 [35]	★	★	★	★	★	★	★		Good
Simonds 2018 [36]	★	★	★	★	★★	★	★		Good
Vendrell 2018 [37]	★	★	★	★	★	★	★		Good
Wang 2022 [38]	★	★	★	★	★★	★	★		Good
Wu 2020 [39]	★	★	★	★	★★	★	★		Good

Characteristics of the Included Studies

Eighteen cohort studies (11 prospective, seven retrospective) included 2790 HIV-positive and 4064 HIV-negative patients; no randomized controlled trials were identified. Most of the studies (15/18) were conducted in sub-Saharan Africa, with others from Brazil, Portugal, and China. The median ages were 43.4 years for the HIV-positive and 54.0 years for the HIV-negative. Squamous cell carcinoma was the predominant histology. Treatment intent was curative in six studies, and mixed (curative/palliative) in three [22,24,27,30-35], whereas it was unspecified in the remaining studies. Table 3 and Table 4 give a comprehensive summary of the demographic and cancer-related characteristics of the included studies.

**Table 3:** Characteristics of the included studies

Study ID	Location	Study design	Study period	Sample size_HIV+	Sample size_HIV-	Age_HIV+ (years)	Age_HIV- (years)	Follow-up_HIV+	Follow-up_HIV-
Dryden-Peterson 2016 [24]	Botswana	Prospective	2010-2015	231	96	41.5 (IQR 37.8-48.4)	57.0 (IQR 48.6-67.7)	30.5 months (0–114 months)	
Ferreira 2017 [25]	Brazil	Retrospective	2001-2013	87	336	<35: 22	<35: 74		
						35-49: 53	35-49: 206		
						50+: 12	50+: 56		
Gichangi 2006 [26]	Kenya	Prospective	2000-2002	41	167	38.1 ± 8.6	50.0 ± 12.1		
Grover 2018 [27]	Botswana	Prospective	2013-2015	96	47	24-39: 36	24-39: 9	633 days (IQR, 348-819 days)	627 days (IQR, 396-885 days)
						40-59: 53	40-59: 26		
						>60: 7	>60: 12		
Grover 2021 [28]	Botswana	Prospective	2013-2018	118	69	46 (IQR 39-50)	61 (IQR 49-70)		
Grover 2022 [29]	Botswana	Prospective	2013-2020	714	311	44 (IQR 39.0-50.0)	61 (IQR 50.5-69.4)	2.2 years (95% CI; 2.0-2.4 years)	2.3 years (95% CI; 2.1-2.7 years)
Grover 2025 [30]	Botswana	Prospective	2015-2019	218	77	44.7	55	44.2 (95% CI; 42.6-46.3) months	
Kavuma 2022 [22]	Uganda	Retrospective	2011-2012	87	134	41.0 (IQR 37.0-47.0)	48.5 (IQR 41.5-55.3)	21.3 months (IQR 7.2-29.1) CFRT 20.5 (7.2-29.4) HFRT 22.1 (7.0-28.7)	21.3 months (IQR 6.3-40.6) CFRT 24.2 (7.4-43.7) HFRT 22.1 (5.4-39.5)
Kigula-Mugambe 2006 [23]	Uganda	Retrospective	2000	7	29	40.7 (range 28-49)	51.9 (range 29-78)	Retrospective study with data follow-up four years	
MacDuffie 2021 [31]	Botswana	Prospective	2013-2015	96	47	43.4	53.9	Median follow-up time for all patients of 37.6 months (63.0 months for living patients)	
Mangena 2015 [32]	South Africa	Retrospective	2007-2008	51	47	43 (SD 11.58)	56 (SD 8.78)		
Meghani 2024 [33]	Botswana	Prospective	2013-2020	789	342	59.1	45.7		
Simonds 2012 [34]	South Africa	Retrospective	2007-2010	59	324	41	50		
Simonds 2015 [35]	South Africa	Prospective	2009-2011	36	177	41 (range 26-62)	50 (range 23-79)		
Simonds 2018 [36]	South Africa	Prospective	2007-2011	71	421	40 (IQR 35-49)	50 (IQR 43-58)		
Vendrell 2018 [37]	Portugal	Retrospective	2012-2016	6	55	48 .0 (IQR 43.2-58.7)	47.9 (IQR 42.5-54.2)		
Wang 2022 [38]	China	Retrospective case-control	2013-2019	30	1289	≥60: 5	≥60: 322		
						40-60: 22	40-60: 842		
						<40: 3	<40: 125		
Wu 2020 [39]	Uganda	Prospective	2013-2015	53	96	44 (IQR 39-48)	54 (IQR 47-62)	All: 14.8 months. Alive and not lost to follow-up: 18.9 months	

**Table 4:** Cervical cancer-related characteristics of the included studies RTOG: Radiation Therapy Oncology Group; CTCAE: Common Terminology Criteria for Adverse Events; RT: radiotherapy/radiation therapy; EBRT: external beam radiation therapy; CT: chemotherapy, CRT: chemoradiotherapy; ICT: intracavitary treatment; HDR: high dose rate

Study ID	HIV+ (Cancer stage I, II, III, IV)	HIV- (Cancer stage I, II, III, IV)	Scale	HIV+ (Histology)	HIV- (Histology)	Treatment_HIV+	Treatment_HIV-	Treatment intent	Treatment doses/ intent
Dryden-Peterson, 2016 [24]	28, 85, 87, 23,	9, 38, 37, 10,	_	Squamous cell - 203, adenocarcinoma - 7, adenosquamous - 4, other/missing - 16	Squamous cell - 81, adenocarcinoma - 8, adenosquamous - 3, other/missing - 4			Curative - 271 Palliative - 47	Treatment intent "as recorded by the treating oncologist"
Ferreira, 2017 [25]	28, 14, 35, 10	111, 56, 134, 35	_	Squamous cell - 81, adenocarcinoma - 6	Squamous cell - 311, adenocarcinoma - 25	First course of treatment RT: 20 RT/CT: 25	First course of treatment RT: 77 RT/CT: 100		
Gichangi, 2006 [26]	1, 26, 13, 1	15, 72, 65, 15	RTOG	Squamous cell - 33, adenocarcinoma - 1, adenosquamous - 0, other - 7	Squamous cell - 148, adenocarcinoma - 10, adenosquamous - 5, other - 4	EBRT	EBRT		Fractionation was 1.8– 2.0 Gy tumor dose daily, five fractions per week for five weeks, with two days of rest from treatment during the weekend
Grover, 2018 [27]	14, 57, 23, 0	3, 24, 18, 2	CTCAE v4.0	Squamous cell - 89, adenocarcinoma - 7	Squamous cell - 41, adenocarcinoma - 6	CT cycles: 4 (3-5) EBRT ≥ 45 Gy: 95 brachytherapy ≥20 Gy: 73	CT cycles: 4 (2-4) EBRT ≥ 45Gy: 45 Brachytherapy ≥20 Gy: 32	Curative	Radical: 45 to 50 Gy whole-pelvis radiation, weekly concurrent cisplatin treatment (35-40 mg/m^2^) for five cycles, and high-dose-rate (HDR) brachytherapy (7 Gy × 3 fractions or 6 Gy × 4 fractions)
Grover, 2021 [28]			_			Brachytherapy - 78 EBRT, boost - 37 CRT - 58	Brachytherapy - 42 EBRT boost - 21 CRT - 32	Curative (Inferred)	RT ± chemotherapy (cisplatin)
Grover, 2022 [29]	123, 268, 248, 59	48, 115, 114, 27	_			RT - 216, CRT - 380, Others: CT - 7, surgery - 43, surgery + CT - 6, surgery + RT - 2, surgery + CRT - 9	RT - 114 CRT - 150 Others: CT - 5, surgery - 14, Surgery + CT - 2, Surgery + RT - 5, Surgery + CRT - 1	Curative (Inferred)	
Grover, 2025 [30]	27, 103, 65, 21	9, 43, 22, 3	CTCAE v5.0	Squamous cell - 67, adenocarcinoma - 2, other - 2	Squamous cell - 67, adenocarcinoma - 2, other - 3	EBRT - 214, brachytherapy - 185, EBRT + brachytherapy - 218, CT - 190	EBRT - 76 Brachytherapy - 68 EBRT + Brachytherapy - 77 CT - 70	Curative	Curative radiation (45-50Gy) to the whole pelvis, followed by high-dose-rate brachytherapy (7 Gy in three fractions or 6 Gy in four fractions) and concurrent weekly cisplatin (40 mg/m^2^)
Kavuma, 2022 [22]	0, 36, 51, 0	0, 50, 84, 0	RTOG	Squamous cell - 82, adenocarcinoma - 1, adenosquamous - 2, other - 2	Squamous cell - 128, adenocarcinoma - 1, adenosquamous - 1, other - 4	EBRT: HFRT - 39, CFRT - 48	EBRT: HFRT - 66 CFRT - 68	Curative	All patients reviewed/presented in this study were treated with curative intent and were given radical doses. Cobalt-60 EBRT dosages of 50 Gy/25# (CFRT) and 45 Gy/15# (HFRT), concurrent chemoradiation received cisplatin of 40 mg/m^2^ once a week, for three to five weeks.
Kigula-Mugambe 2006 [23]	0, 2, 4, 0	2, 7, 18, 2	RTOG			EBRT - ICT - 4	EBRT - ICT - 18		EBRT 50-60 Gy in 25-30 fractions. One month after EBRT, brachytherapy 20-30 Gy (ICT)
MacDuffie, 2021 [31]	14, 57, 23, 0	3, 24, 18, 2						Curative	45 to 50 Gray (Gy) of whole pelvis RT, weekly concurrent cisplatin (35–40 mg/m^2^) for five cycles, and high-dose-rate brachytherapy (7 Gy in three to four fractions or 6 Gy in four to five fractions)
Mangena, 2015 [32]	I-IIA - 5, IIB-IIIA - 21, IIIB-IVA - 25	I-IIA - 5, IIB-IIIA - 15, IIIB-IVA - 27	RTOG	Squamous cell - 49, non-squamous - 2	Squamous cell - 43, non-squamous - 4	Curative - 44, palliative - 7, EBRT - 51, external boost - 8, brachytherapy - 29, concurrent CT - 38	Curative - 43, palliative - 4, EBRT - 47, external boost - 6, brachytherapy - 32, concurrent CT - 34	Curative/ Palliative	Curative: 46 Gy for stage IIB and 50 Gy for stages III and IV in 2 Gy fractions, followed by high-dose-rate therapy of 26 Gy in four fractions, and 24 Gy in three fractions, respectively. Palliative: 8-30 Gy, administered between one and 10 fractions. Adjuvant postoperative therapy: radiation 45 Gy in 25 fractions, with the addition of high-dose-rate treatment (20 Gy in four fractions) and chemotherapy.
Meghani, 2024 [33]	97, 208, 177, 122	42, 103, 92, 40	_	Squamous cell - 67, adenocarcinoma - 2, other - 2	Squamous cell - 67, adenocarcinoma - 2, other - 3	Curative - 330, RT only: high-dose/definitive RT - 123, low-dose/palliative RT - 107, palliative - 12	Curative - 135, RT only: high-dose/definitive RT - 75 low dose/palliative RT - 47, palliative - 5	Curative/palliative	Curative chemoradiation treatment was defined as cisplatin chemotherapy + RT. Palliative RT was defined as low-dose RT only (EBRT <4000 cGy and no brachytherapy). Definitive RT alone was defined as high‐dose RT only (external beam radiation therapy (EBRT) ≥ 4000 cGy and any brachytherapy).
Simonds, 2012 [34]	0, 10, 49, 0	6, 103, 215, 0	_	Squamous cell - 56, adenocarcinoma - 1, other - 16	Squamous cell - 300, adenocarcinoma - 15, other/missing - 9	Curative - 49 (completed - 22)	Curative - 284 (completed - 199)	Curative	Stage IB2 to IIIB cervical carcinoma is 46 to 50 Gy in 23 to 25 fractions of EBRT delivered to the pelvis with concurrent weekly cisplatin chemotherapy at a dose of 40 mg/m^2^ for four to six cycles and HDR brachytherapy at 20 to 26 Gy in four to five fractions starting in week 5 of EBRT.
Simonds, 2015 [35]	IB - IIIA- 9 IIIB - 27	IB - IIIA- 56 IIIB - 121	RTOG	Squamous cell - 34, adenocarcinoma - 1, other - 1	Squamous cell - 163, adenocarcinoma - 5, other - 9	EBRT - 14, CRT - 22	EBRT - 43, CRT - 134	Curative	46-50 Gy external beam radiation in 23-25 fractions followed by 20–25 Gy high-dose-rate intracavitary brachytherapy in four to five fractions ± 40 mg/m^2^ cisplatin weekly. Due to pressure on the radiotherapy waiting list, in some cases of Stage IIIB disease, chemotherapy was omitted, and a hypofractionated regimen of 40.05 Gy in 15 fractions, or 42.72 Gy in 16 fractions, was used with standard HDR brachytherapy.
Simonds, 2018 [36]	IIB - 15 III - 56	IB1-IIA - 12, IIB - 120, III - 289	_	Squamous cell - 67, adenocarcinoma - 2, other - 2	Squamous cell - 391, adenocarcinoma - 15, other - 15	EBRT - 64, Hypo - 7	EBRT - 369, Hypo - 52	Curative (inferred)	Standard fractionation/EBRT - 45 to 52.5 Gy in 23 to 28 fractions. Hypofractionation - 40.05 Gy in 15 fractions, or 42.72 Gy in 16 fractions, and no chemotherapy.
Vendrell, 2018 [37]	0, 2, 3, 1	0, 28, 22, 5	CTCAE v4.0					Curative (inferred)	Cisplatin + whole pelvic RT (at least 45Gy in 25 fractions) + EBRT (45–50.4Gy in 25–28 fractions)/ brachytherapy (24 Gy/4 fractions or 21 Gy/3 fractions) boost
Wang, 2022 [38]	I-II - 15 III-IV - 15	I-II - 1083 III-IV - 206	_	Squamous cell - 22, other - 8	Squamous cell - 1020, other - 269	Non-surgical - 21, surgery - 9	Non-surgical - 300, surgery - 989	Curative (inferred)	
Wu, 2020 [39]	10, 26, 15, 2	12, 47, 33, 4	_	Squamous cell - 48, adenocarcinoma - 4, other - 1	Squamous cell - 95, adenocarcinoma - 4, other - 1	Initial treatment CRT - 10, RT only - 27, Others: CT - 1, surgery only - 1, surgery + RT - 1, surgery + CT - 1	Initial treatment CRT - 21, RT only - 40, Others: CT - 1	Curative (inferred)	45 Gy of external-beam radiation delivered over 15 fractions, followed by 25 Gy of low-dose rate brachytherapy delivered in one fraction

HIV-Related Characteristics

**Figure 2:**
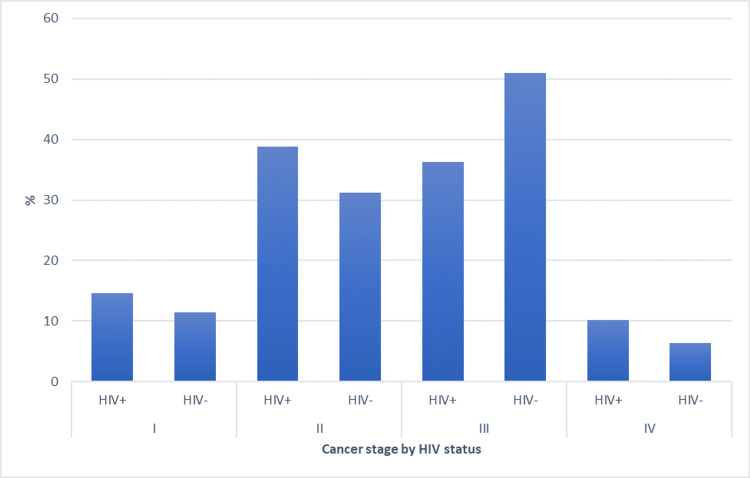
Prevalence of HIV-positive cases with cervical cancer and presentation as per cancer staging

Eleven studies reported the number of patients on ART, which ranged from 32% to 100% [24,25,27-31,34-37]. Of these, seven studies reported the specific ART drugs or drug groups used in the regimens specified; these included stavudine/tenofovir, lamivudine, efavirenz, and dolutegravir-based combinations [24,27,28,34-37]. Median CD4 counts ranged from 263 to 567.5 cells/µL [23-28,30,32-34,36,39]. HIV-positive patients had a higher proportion of stage II disease, while HIV-negative patients had more stage III disease (Figure 2). HIV-related characteristics are summarized in Table 5.

**Table 5:** HIV-related characteristics of the included studies

Study ID	Sample size	Initiation of ART	ART regimen	No. on ART	CD4 counts	Timing of CD4 counts	Viral load
Dryden-Peterson 2016 [24]	231	Before cancer diagnosis - 189 (81.8%) (median ART duration 4.8 years; IQR, 1.6 to 8.6 years) Cancer was initial ART-qualifying condition - 14 (6.1%)	tenofovir disoproxil fumarate/emtricitabine/efavirenz or nevirapine - 107 zidovudine/lamivudine/efavirenz or nevirapine - 74 Second line - 13 Unknown ART - 19 No ART - 18	213	397 cells/μl (264-555) ; 24 patients (10.4%) - < 200 cells/mL	"Recent CD4"	HIV RNA< 1,000 copies/mL - 133 (97.1%)
Ferreira 2017 [25]	87	55 (63%) received HAART at some point during care		55	263 cells/μl (IQR 137-368)	"closest to treatment"	
Gichangi 2006 [26]	41						
Grover 2018 [27]	96	At presentation for CRT, "96% (92 of 96) of the HIV-infected patients had already been taking ART for a median of 84 months (IQR, 24-120 months)." Rest - started ART before the start of cancer treatment	tenofovir, emtricitabine, and efavirenz	96	481 cells/μl (IQR 351-579)		
Grover 2021 [28]	118	110 (93%) on ART at cancer diagnosis	efavirenz, tenofovir disoproxil fumarate, and emtricitabine OR dolutegravir–tenofovir disoproxil fumarate and emtricitabine	110	445 cells/ml (IQR 319–657)		≥400 cells/ml - 11 <400 cells/ml - 75
Grover 2022 [29]	714	676 women (94.7%) were receiving antiretroviral therapy at the time of cervical cancer diagnosis		676	429.5 cells/μl (240.0-619.5)		≥400 cells/ml - 91 <400 cells/ml - 396
Grover 2025 [30]	218	96.3% were on anti- retroviral therapy		210	baseline - 470 cells/μL; CD4 < 350 cells/μL - 97.3% at end of treatment and 94.8% 3 months post-treatment	baseline, end of treatment, and 3 months post-treatment	undetectable baseline - 81.8%
Kavuma 2022 [22]	87				444 cells/mm^3^ (210-880)		
Kigula-Mugambe 2006 [23]	7				289 cells/ml (± 122 SD)		
MacDuffie 2021 [31]	96	Before cancer treatment initiation. Median time on ART of 84 months (IQR, 24–120 months)		96	481 cells/μL (IQR, 351-579 μL)		
Mangena 2015 [32]	51	"Many patients were first diagnosed with HIV infection at the time of cervical cancer diagnosis, and therapy for both diseases was initiated at the same time."					
Meghani 2024 [33]	789				473 cells/μl (321–665)		
Simonds 2012 [34]	59	At initial evaluation - 19	stavudine/tenofovir, lamivudine, and efavirenz	19	354 cells/μL (range, 33-1249)		
Simonds 2015 [35]	36	Before RT - 16 During or after RT - 20	lamivudine, efavirenz and tenofivir/stavudine	36	341 cells/μl (range 33–790)		
Simonds 2018 [36]	71	43 - before cancer diagnosis 28 -before or during EBRT	lamivudine, efavirenz and tenofivir/stavudine	71	All: 386 cells/μl (IQR 256–450); 43 patients: 366 cells/μl (IQR 276–458) 28 patients: 324 cells/μl (iQR 209–407)		
Vendrell 2018 [37]	6	ART during CRT - 4 No ART - 2	non-nucleoside reverse transcriptase inhibitor (NNTRI) - 1; nucleoside reverse transcriptase inhibitors (NTRI) + protease inhibitors (PI) - 2 ; NTRI + Integrase inhibitor (II) - 1	4	567.5 cells/mm^3^ (range 59.9–1181.6) On ART - mean 747.8 cells/mm^3^ (95% CI; 271.2 - 1324.3) Not on ART - mean 69.3 cells/mm^3^ (95% CI; 163.9 - 302.4)	Before CRT	
Wang 2022 [38]	30			30		"every 3 months in the first year and twice a year from the second year"	
Wu 2020 [39]	53			41	373 cells/mm^3^ (IQR 300–502)	at enrolment	≥500 copies/mL - 12 < 500 copies/mL - 41

Survival Outcomes

Eleven studies reported OS. The one-year OS was 65-90% for the HIV-positive group and 69-99% for the HIV-negative group [23,38,39] (Table 6). The two-year rates were 30-86.7% and 41.6-92.6% [23,27-29,31,33,36,39], and the five-year rates were 44.4-65.0% and 49.2-69.9% [22,29,31,36,38], respectively.

**Table 6:** Overall survival for the included studies OS: overall survival; CRT: chemoradiotherapy; RT: radiotherapy; CFRT: conventional fractionated radiotherapy; HFRT: hypofractionated radiotherapy

	OS_median		OS_%	
Study ID	HIV+	HIV-	HIV+	HIV-
Dryden-Peterson 2016 [24]			3-yr: 35% (27%-44%)	3-yr: 48% (35-60%)
Ferreira 2017 [25]				
Gichangi 2006 [26]				
Grover 2018 [27]			2-yr: 65% (95% CI; 54%-74%)	2-yr: 66% (95% CI; 49%-79%)
Grover 2021 [28]	21.5 months (95% CI 12.9 to 30.1 months) CRT: 57.3 months (95% CI 16.1 to 98.6 months) RT: 15.2 months (95% CI 10.6 to 19.8 months)	20.3 months (95% CI 16.6 to 44.0 months) CRT: 21.0 months (95% CI 5.1 to 36.8 months) RT- 21.0 months (95% CI 5.1 to 36.8 months)	2-yr CRT: 59.8±6.5% 2-yr RT: 38.3±6.3%	2-yr CRT: 58.9±8.8% 2-yr RT: 45.5±8.2%
Grover 2022 [29]			2-yr: 65.9% (95% CI; 62.4%–69.6%) 5-yr: 60% (95% CI; 50.8%–59.5%)	2-yr: 71.6% (95% CI; 66.6%–76.9%) 5-yr: 60.5% (95 % CI; 54.8%–66.9%)
Grover 2025 [30]				
Kavuma 2022 [22]			30.0% CFRT 5-yr: 27.7% HFRT 5-yr: 30.7%	44.9% CFRT 5-yr: 45.7% HFRT 5-yr: 44.2%
Kigula-Mugambe 2006 [23]			1-yr: 67% 2-yr: 40% 3-yr: 27% 4-yr: 0%	1-yr: 89% 2-yr: 62% 3-yr: 51% 4-yr: 46%
MacDuffie 2021 [31]			2-yr: 65.0% (95% CI; 54.0-74.0) 5-yr: 55.1% (95% CI; 44.2%–64.7%)	2-yr: 66.0% (95% CI; 49.0-79.0) 5-yr: 56.8% (95% CI; 40.0%–70.5%)
Mangena 2015 [32]	21 months	32 months		
Meghani 2024 [33]	Any treatment group - 5.5 years Curative CRT - not reached Definitive RT - 3.6 years Palliative RT - 1.6 years	Any treatment group - not reached Curative CRT - not reached Definitive RT - not reached Palliative RT - 1.4 years	2-yr: Any treatment group - 76.5% Curative CRT - 86.7% Definitive RT - 75.2% Palliative RT - 38.2%	2-yr: Any treatment group - 79.9% Curative CRT - 86.9% Definitive RT - 92.6% Palliative RT - 41.6%
Simonds 2012 [34]				
Simonds 2015 [35]				
Simonds 2018 [36]			2-yr: 41.6% (95% CI; 29.5%–53.7%) 5-yr: 35.9% (95%CI; 23.9%–48.0%) 5-yr: CRT: 35.2%, RT: 20.2%	2-yr: 62.0% (95%CI; 57.2%–66.7%) 5-yr: 49.2% (95%CI; 44.6%–54.4%) 5-yr: CRT: 57.0%, RT: 36.2%
Vendrell 2018 [37]				
Wang 2022 [38]	59 months	83 months	1-yr: 90.0% 3-yr: 73.3% 5-yr: 44.4%	1-yr: 99.0% 3-yr: 91.0% 5-yr: 69.6%
Wu 2020 [39]	14.7 months	24.3 months	1-yr: 65% (95% CI; 51%–77%) 2-yr: 30% (95% CI; 17%–44%)	1-yr: 69% (95% CI; 58%–77%) 2-yr: 51% (95% CI; 39%–62%)

**Figure 3:**
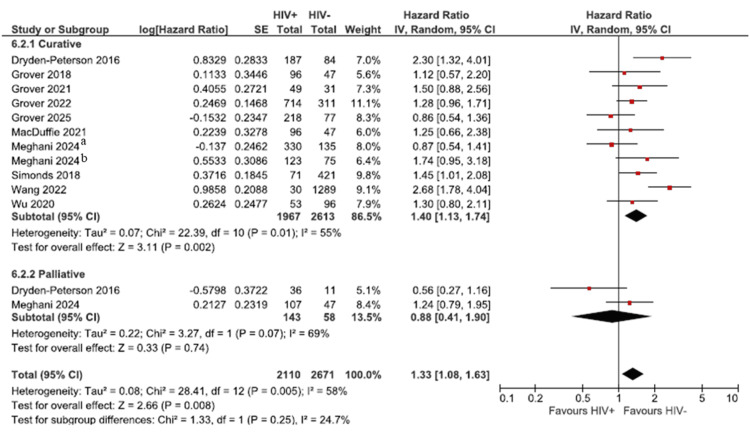
Pooled overall survival rates for the HIV-negative and HIV-positive groups The figure illustrates the pooled overall survival by treatment intent.  This was calculated in RevMan using the generic inverse variance. The hazard ratio (HR), upper and lower values of the 95% confidence interval, or the p-value were entered. ^a^Curative CRT arm; ^b^definitive RT. Dryden-Peterson 2016 [24], Grover 2018 [27], Grover 2021 [29], Grover 2022 [29], Grover 2025 [30], MacDuffie 2021 [31], Meghani 2024 [33], Simonds 2018 [36], Wang 2022 [38], Wu 2020 [39]

Ten studies reported the effect of HIV on the OS with a pooled population of 2,110 HIV-positive patients and 2,671 HIV-negative patients [24,27-31,33,36,38,39]. The meta-analysis showed that HIV-positive individuals had significantly worse OS compared with HIV-negative individuals beneficial association of OS in the HIV-negative population. The pooled HR for OS was 1.33 (95% CI 1.08-1.63), indicating a statistically significant 33% increased risk of death in HIV-positive patients (Figure 3). Some studies reported the HR only by treatment intent, so we created subgroups by treatment intent, as stated by the authors or inferred from the treatment regimens. Curative-intent treatment reported an HR of 1.40 (95% CI 1.13-1.74), suggesting that among patients receiving curative therapy, HIV-positive patients experienced significantly worse OS than HIV-negative patients. The heterogeneity within this subgroup was moderate (I² = 55%, p = 0.01), indicating some variability across the studies. Upon excluding the only non-sub-Saharan study [38], heterogeneity was substantially reduced to I² = 22%, indicating much improved consistency among the remaining studies. This exclusion did not change the direction (HR 1.29; 95% CI 1.09-1.54) or statistical significance of the effect. The palliative subgroup had an HR of 0.88 (95% CI: 0.41-1.90) with high but non-significant heterogeneity (I² = 69%, p = 0.07). The effect was non-significant and imprecise, likely reflecting smaller study numbers and clinical variability.

**Figure 4:**
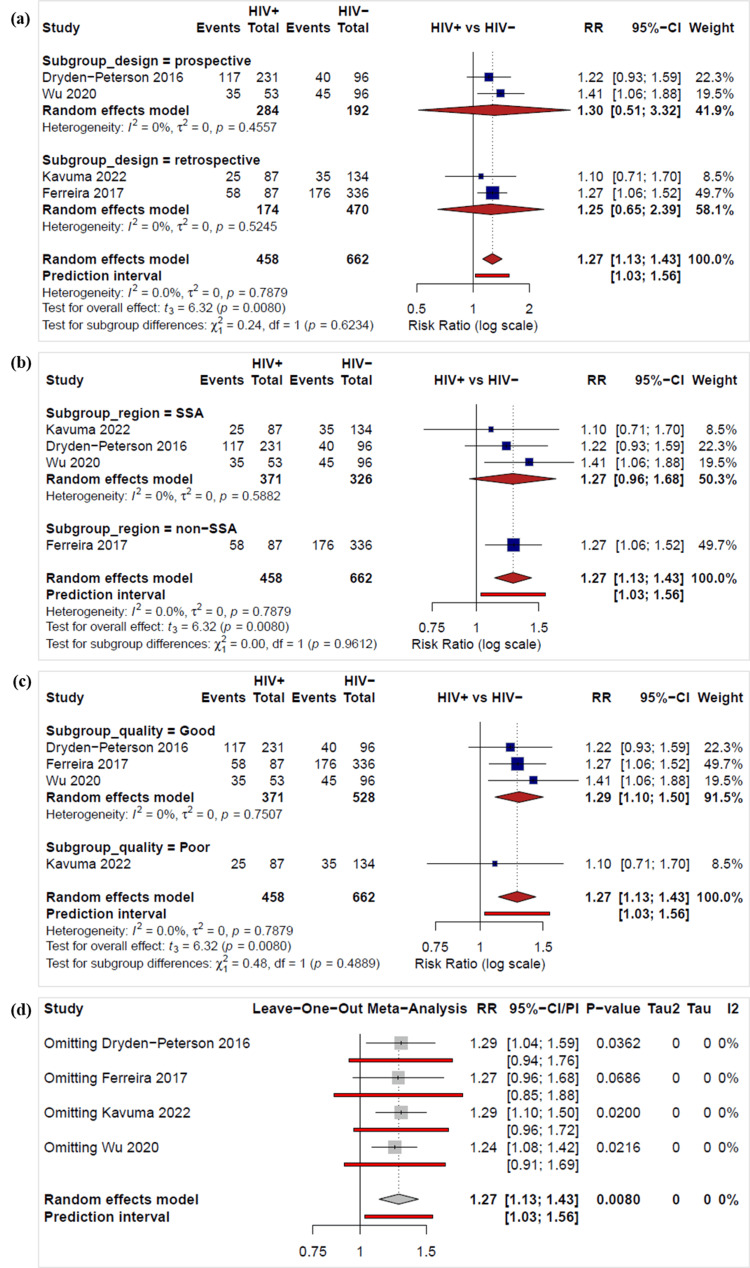
Forest plots of the risk ratios of deaths between the HIV-negative and HIV-positive groups by (a) study design, (b) study region, (c) study quality subgroups, and (d) a leave-one-out sensitivity analysis The calculations were done in R version 4.4.1 (see Appendix B for R code). Dryden-Peterson 2016 [24], Ferreira 2017 [25], Grover 2018 [27], Grover 2021 [28]

The risk of death was higher in the HIV-positive group compared to the HIV-negative group (Figure 4) [22,24,25,39]. However, the confidence intervals crossed 1 or came close to crossing it, indicating uncertainty in individual study estimates. The pooled risk ratio (random-effects model) was 1.27 (95% CI 1.13-1.43), indicating that HIV-positive individuals have a 27% higher risk of death compared to HIV-negative individuals. The studies showed no heterogeneity (I² = 0%). There were no differences in subgroup analyses by study design (Figure 4a), geographical location (sub-Saharan Africa vs non-sub-Saharan Africa study sites) (Figure 4b), or study quality (Figure 4c). 

A leave-one-out sensitivity analysis produced pooled risk ratios ranging from 1.24 to 1.29 (Figure 4d). All the pooled effect estimates remained statistically significant except when Ferreira 2017 [25] was omitted (p = 0.0686). The corresponding 95% CIs ranged from [1.08, 1.42] to [1.10, 1.50], and all analyses showed no heterogeneity (I² = 0%). The overall random-effects meta-analysis estimate was RR = 1.27 (95% CI 1.13-1.43, p = 0.0080). Omitting Kavuma 2022 [22], which was rated poor quality, did not change the direction (HR 1.29; 95% CI 1.13-1.47) or statistical significance of the effect.

**Figure 5:**
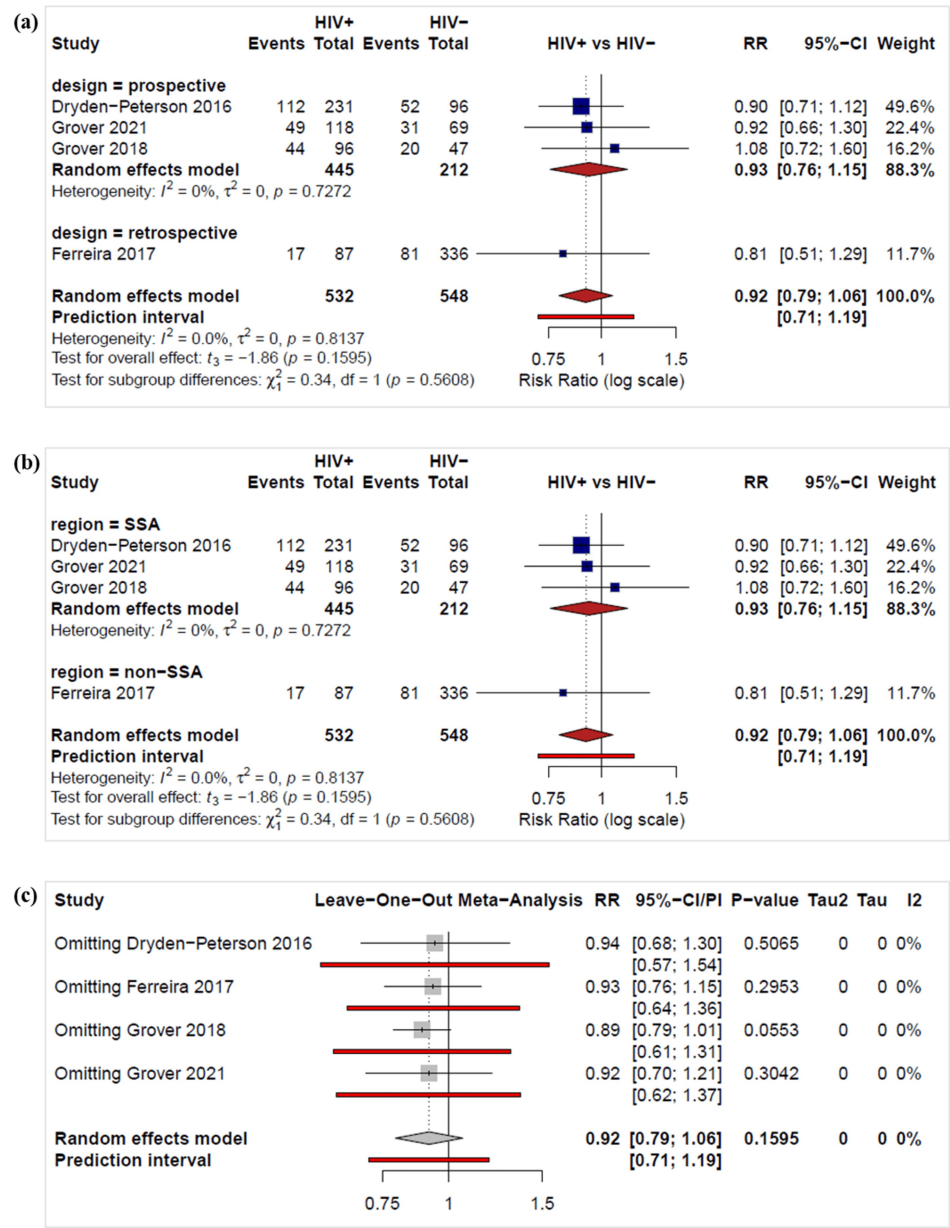
Forest plots of the risk ratios of complete response between the HIV-negative and HIV-positive groups by (a) study design, (b) study region subgroups, and (d) a leave-one-out sensitivity analysis. The calculations were done in R version 4.4.1 (See Appendix B for R code). Dryden-Peterson 2016 [24], Ferreira 2017 [25], Grover 2018 [27], Grover 2021 [28].

Meta-analysis of complete response rates showed no significant difference between HIV-positive and HIV-negative individuals by study design (Figure 5a) or study region (Figure 5b) [24,25,27,28]. The pooled risk ratio (random-effects model) was 0.92 (95% CI: 0.79-1.06), indicating that HIV-positive patients were slightly less likely to achieve a complete response; however, this difference was not statistically significant as the confidence interval crossed 1. Individual studies demonstrated similar findings, with all confidence intervals overlapping 1. Heterogeneity across studies was negligible (I² = 0%), indicating strong consistency in the direction and magnitude of effect.

Excluding each study sequentially in a leave-one-out sensitivity analysis yielded pooled risk ratios ranging from 0.89 to 0.94, none of which were statistically significant (all p-values > 0.05) (Figure 5c). The corresponding 95% CIs were consistently wide, and all crossed the null value of 1, indicating no statistically significant association regardless of which study was removed. In addition, no heterogeneity was detected (I² = 0%). The overall random-effects model produced an RR of 0.92 (95% CI 0.79-1.06, p = 0.1595).

Treatment Toxicities

Eight studies reported toxicities using different scales: five used RTOG [22,23,26,32,35], one used CTCAE v5.0 [30], and two used CTCAE v4.0 [27,37] (Table 7). Three studies reported toxicities but did not indicate the grading scale [24,32,38]. Toxicity results were reported differently, even for studies that used a grading scale. In most studies, it was unclear whether the reported results were the number of patients who experienced at least one event or the total number of events; therefore, the overall toxicity rate could not be calculated. Four studies reported no difference in toxicities by HIV status [22,24,27,30,38]; five studies reported differences, one of which showed differences only with ≥ grade 3 toxicities [26,32,35,37,38]. Furthermore, toxicities were not reported in relation to radiation doses, radiotherapy versus chemotherapy, or concurrent ART use; hence, the impact of these treatment modalities was also not assessed.

**Table 7:** Summary of reported hematological, skin, genitourinary, and gastrointestinal toxicities in HIV-negative and HIV-positive groups RTOG: Radiation Therapy Oncology Group; CTCAE: Common Terminology Criteria for Adverse Events; NI: not indicated; Hb: hemoglobin; WBC: white cell count

	Scale	Hematological			Skin			Genitourinary			Gastrointestinal		
Study ID		Grade	HIV+	HIV-	Grade	HIV+	HIV-	Grade	HIV+	HIV-	Grade	HIV+	HIV-
Dryden-Peterson 2016 [24]		NI	1	0	NI	102	38	NI	80	38	NI	120	58
Ferreira 2017 [25]													
Gichangi 2006 [26]	RTOG				3 to 4	16	54	3 to 4	8	8	3 to 4	14	69
Grover 2018 [27]	CTCAE v4.0					52	27		6	3		52	26
Grover 2021 [28]													
Grover 2022 [29]													
Grover 2025 [30]	CTCAE v5.0	≥ 2	Neutrophil count - 70 WBC count - 130 Hb - 84	Neutrophil count - 22 WBC count - 49 Hb - 24	≥ 2	Radiation dermatitis - 53	Radiation dermatitis - 15	≥ 2	Urinary incontinence - 0; urinary urgency - 1; pelvic pain - 5; vaginal discharge - 7; vaginal hemorrhage - 20	Urinary incontinence - 2; urinary urgency - 1; pelvic pain - 4; vaginal discharge - 3; vaginal hemorrhage - 6	≥ 2	nausea - 4; vomiting - 1; diarrhea - 8	nausea - 2; vomiting - 3; diarrhea - 3
Kavuma 2022 [22]	RTOG												
Kigula-Mugambe 2006 [23]	RTOG												
MacDuffie 2021 [31]													
Mangena 2015 [32]	RTOG	NI	1	1	NI	12	4	NI	6	3	NI	0	1
Meghani 2024 [33]													
Simonds 2012 [34]													
Simonds 2015 [35]	RTOG	3 to 4 2	18 42	37 136	3 to 4	1	2	3 to 4	0	1	3 to 4	3	23
Simonds 2018 [36]													
Vendrell 2018 [37]	CTCAE v4.0	1 to 2 3-4	neutropenia 4 1	neutropenia 19 2									
Wang 2022 [38]		NI	29	947				NI	3	67	NI	15	670
Wu 2020 [39]													

Discussion

This systematic review of 18 cohort studies provides critical insights into the compounded challenges faced by HIV-positive women with cervical cancer undergoing radiotherapy or chemoradiation, particularly in LMICs [13,40]. The findings highlight significantly poorer survival outcomes and a lower likelihood of complete response in this population, possibly driven by a complex interplay of biological, clinical, and systemic factors. However, treatment-related toxicities show conflicting results due to variations in the methodologies used for the collection of toxicity data between studies. 

Survival Outcomes

HIV-positive women exhibited lower OS rates compared to HIV-negative counterparts (HR 1.33 (95% CI 1.08-1.63)), consistent with prior meta-analyses [3,4,13]. This disparity with HIV status likely stems from HIV-related immunosuppression, which impairs immune surveillance and exacerbates human papillomavirus (HPV) persistence, a key driver of cervical carcinogenesis [3-5,41]. The synergistic interaction between HIV and HPV accelerates disease progression, as HIV-induced CD4 depletion enhances HPV replication and integration into host DNA, leading to higher rates of invasive cancer [4,5]. However, the HPV status of the patients in the included studies was not reported. Furthermore, HIV-positive patients often present with advanced-stage disease (stage II-III), as observed in our review, which further compromises survival [24,39].

Beyond biological factors, systemic barriers significantly contribute to poorer outcomes. In LMICs, where 15 of the 18 studies were conducted, limited access to screening, delayed diagnosis, and inadequate healthcare infrastructure exacerbate mortality risk [40,42,43]. For instance, studies from sub-Saharan Africa reported lower survival rates than those from high-income settings, reflecting disparities in diagnostic tools, radiotherapy availability, and supportive care [27,40]. Grover et al. highlighted that robust healthcare systems in high-income countries facilitate earlier detection and comprehensive treatment, significantly improving prognosis [27]. In contrast, logistical challenges in LMICs, such as transportation barriers and treatment interruptions, further widen the survival gap [43,44]. The higher HR for disease-free survival (DFS; HR 1.45, 95% CI 1.20-1.75) and increased recurrence rates in HIV-positive patients underscore the need for intensified post-treatment surveillance, particularly in resource-limited settings where follow-up care is often inconsistent [7,13].

Treatment Toxicities

Due to inconsistencies in the reporting style of toxicities, we were not able to compare these between HIV-negative and HIV-positive individuals. There is conflicting evidence on radiation-based treatment toxicities in women with cervical cancer by HIV status. Some studies have shown differences, while others have shown minimal differences in toxicities in HIV-positive patients compared to HIV-negative [13,45]. Toxicities are multifactorial and can be driven by HIV-related bone marrow suppression, drug-drug interactions between ART and cisplatin-based chemoradiation, and baseline immune dysfunction [9,46]. For instance, protease inhibitors (PIs) can inhibit cytochrome P450 enzymes, increasing cisplatin toxicity, whereas integrase strand transfer inhibitors (INSTIs) have a lower interaction profile, resulting in fewer adverse events [37,47,48]. This suggests that ART regimens with fewer drug-drug interactions, such as INSTI-based regimens, are biologically plausible options to reduce interactions with cytotoxic therapy; however, prospective data are needed. Since the studies included in this review did not report on the effect of ART use on the clinical outcomes and toxicities in patients treated with radiation-based therapies, this was not evaluated in the review.

The elevated toxicity burden in LMICs is particularly concerning, as limited access to advanced radiotherapy techniques, such as intensity-modulated radiotherapy (IMRT) or image-guided radiotherapy (IGRT), increases normal tissue exposure and toxicity [49]. Studies from sub-Saharan Africa reported the highest toxicity rates, compounded by inadequate supportive care (e.g., Granulocyte colony-stimulating factor (GCSF) and antiemetics) and inconsistent ART adherence [50]. In contrast, high-resource settings that leverage IMRT/IGRT achieve lower toxicity rates while maintaining efficacy, highlighting the potential of technology transfer to LMICs [51,52]. Strategies to mitigate toxicities, such as prophylactic granulocyte colony-stimulating factors or dose-adjusted chemoradiation protocols, warrant further exploration, particularly for patients with low CD4 counts (<200 cells/µL), who are at higher risk [37,53]. Additionally, patient education and adherence support programs could reduce treatment interruptions, a common issue in resource-constrained settings [42].

Prognostic Factors

The strong correlation between higher CD4 counts (>200 cells/µL) and improved survival underscores the critical role of immune function in treatment response [12,27,54]. CD4 counts serve as a robust prognostic marker, with counts above this threshold associated with reduced mortality risk [27]. This finding highlights the importance of initiating early ART to restore immune function before cancer treatment [27,55]. Emerging biomarkers, such as HPV viral load and immune checkpoint expression, also show promise in predicting outcomes [55,56]. High HPV viral load has been linked to worse prognosis in HIV-positive patients, potentially due to enhanced oncogenic activity, suggesting a role for biomarker-driven risk stratification [56]. However, the clinical utility of these biomarkers remains limited by inconsistent measurement standards and a lack of validation in LMIC settings [57,58].

Socioeconomic and systemic factors further influence prognosis. In LMICs, poverty, stigma, and limited healthcare access delay diagnosis and treatment, contributing to advanced disease at presentation [43,44]. Gender-specific barriers, such as caregiving responsibilities and lack of autonomy, disproportionately affect women, exacerbating disparities [59]. Community-based interventions, as described by Tapela et al. in Rwanda, demonstrate that task-shifting and mobile health technologies can improve access to care and should be scaled up [60,61]. Integrating cervical cancer screening into HIV care programs could also facilitate earlier detection, particularly in high-prevalence regions [43,62].

Implications for Clinical Practice

The intersection of HIV and cervical cancer in resource-limited settings necessitates targeted interventions for cervical cancer. The findings of this review advocate for a multidisciplinary approach integrating oncology, infectious disease, and public health expertise. Clinicians should prioritize early ART initiation and CD4 monitoring to optimize immune status before cancer treatment [27,63]. Selecting ART regimens with lower toxicity profiles, such as INSTIs, can reduce adverse events and improve treatment completion rates [37,64]. In LMICs, investments in radiotherapy infrastructure (e.g., IMRT, IGRT) and supportive care (e.g., prophylactic growth factors, nutritional support) are crucial for reducing toxicities and improving quality of life [51,53]. Enhanced screening programs targeting HIV-positive women, such as HPV-based testing integrated into HIV clinics, could facilitate earlier detection and improve outcomes [43].

Policy interventions are equally vital. National cancer control plans in LMICs should prioritize HIV-positive populations, with funding allocated for advanced radiotherapy and training for healthcare providers [51,65]. Successful models, such as Botswana’s cervical cancer program, which integrates HIV and oncology care, offer a blueprint for other LMICs [31,54]. Telemedicine and community health worker programs can bridge gaps in rural areas, ensuring continuity of care [44,66]. These strategies, combined with patient-centred approaches addressing stigma and adherence, could significantly reduce the burden of cervical cancer in this population.

Research Gaps and Future Directions

Despite these insights, significant research gaps persist. The predominance of retrospective studies in this review limits data consistency and the ability to control for confounding factors, such as age, performance status, comorbidities, ART duration, and cancer stage. Recent trends indicate a demographic shift in cervical cancer incidence toward younger populations [67]. Evidence suggests that these younger patients often experience poorer outcomes within the HIV care continuum [68]. This underscores the necessity for further exploration into the implications and outcomes of cervical cancer in conjunction with HIV management in this age group. Survival is significantly influenced by factors such as cancer stage and performance status [69]. Moreover, studies have shown that early and sustained ART use correlates with a decrease in high-risk HPV persistence and a reduction in the incidence and progression of precancerous lesions [70]. Failing to account for these variables introduces bias into the interpretation of study results. Future prospective studies that integrate these confounding factors are essential for enhancing patient management strategies for those facing the dual challenges of cervical cancer and HIV.

Inconsistent reporting of ART regimens, toxicity grades, and long-term outcomes hinders comprehensive meta-analyses. Prospective, multicenter trials are urgently needed to standardize data collection and assess the interplay between ART, cancer treatments, and patient outcomes. Such trials should include diverse settings to ensure global applicability, as the underrepresentation of high-resource settings in this review restricts comparative insights [13].

Implementation science provides a promising framework for addressing barriers specific to LMICs. Studies have demonstrated the efficacy of task-shifting, telemedicine, and community-based care in improving access, but scalability remains a challenge [44,61]. Research into cost-effective radiotherapy solutions, such as hypofractionated regimens, could enhance feasibility in resource-limited settings [22]. Biomarker-driven therapies, including HPV viral load and immune checkpoint inhibitors, warrant further exploration [57]. For instance, immune checkpoint inhibitors, which enhance antitumor immunity, may be particularly effective in HIV-positive patients with high tumor mutational burden, though safety in this population requires investigation [57,71]. Finally, the role of the cervical microbiota in cancer risk, as highlighted by Klein et al., suggests a novel avenue for preventive strategies [41].

Global health perspectives are critical. Collaborative initiatives, such as those supported by the International Atomic Energy Agency, can facilitate technology transfer and capacity building in LMICs [8]. Partnerships between high- and low-resource settings could accelerate research and implementation, reducing disparities in outcomes. Addressing these gaps through targeted, interdisciplinary research will be essential to improving survival and quality of life for HIV-positive women with cervical cancer.

Limitations

The calculation of pooled OS by year was not feasible because, although survival rates were reported for various years across the studies, the HRs were presented as overall estimates for each study. Furthermore, ART reporting was inconsistent across studies. In addition, restricting our literature search to English-language publications may have introduced a language bias, potentially omitting relevant studies published in other languages. Notably, a significant proportion of the studies included in our analysis were conducted in sub-Saharan Africa, which may limit the applicability of our findings to high-resource settings. There was also potential for survivorship bias, where initial analysis did not evaluate all patients; for instance, where incompletely treated patients or those with incomplete data were excluded from initial analysis leading to skewed or inaccurate conclusions. 

## Conclusions

This systematic review emphasizes the effects of ART on the outcomes and toxicities of radiation-based therapies for cervical cancer in HIV-infected patients. Women with HIV and cervical cancer experience lower survival rates and lower complete response, but there are mixed findings on toxicities, especially in LMICs. However, existing studies do not provide detailed information on these outcomes by specific treatment methods, such as radiation dose, differences between radiotherapy and chemotherapy, or the use of concurrent ART. Future investigations must prioritize bridging existing knowledge gaps in the field. It is crucial to implement prospective multicenter studies and ART-stratified trials that utilize standardized toxicity assessment protocols. Additionally, controlling for confounding variables, such as patient age, disease stage, performance status, and comorbidities- is necessary. This approach will enhance our understanding of the influence of ART on both clinical outcomes and toxicity profiles in cervical cancer patients undergoing radiation-based therapies. By tackling these complexities through focused research and informed policy initiatives, we can improve management strategies for this vulnerable patient population.

## Appendices

Appendix A: review protocol

Cervical Cancer Outcomes and Toxicity in HIV-Positive Women Treated with Radiation or Chemoradiation: A Systematic Review

Review Question:

What is the effect of HIV status on clinical outcomes, toxicities and prognostic factors in women undergoing radiotherapy or chemoradiation for cervical cancer?

Condition or Domain Being Studied

Cervical cancer

Rationale for the Review

Treating cervical cancer in HIV positive patients requires a better understanding of the outcomes and the associated risk factors, and also an update on the toxicity profile associated with this disease population. Many factors need to be assessed regarding disease and treatment characteristics that impact disease outcome, and the toxicities associated with this disease population need to be defined. This review aims to assess these factors, which may help refine treatment delivery and thereby better understand survival outcomes and assess treatment-related toxicities.

Review Objectives

To evaluate the treatment outcomes, patterns of treatment failure, and risk factors in early and locally advanced cervical cancer in patients with HIV positive disease treated with radiation therapy (RT) alone or concurrent chemoradiation therapy.

Keywords

Cervical cancer, radiation therapy, chemoradiation,

Eligibility Criteria

Study design: Relevant studies, including case-control, retrospective cohort, prospective cohort, and randomized controlled trials, will be included in this study. Case reports, case series, critical reviews, systematic reviews, and meta-analyses will be excluded.

Population (P): Adult HIV+ and HIV- patients (≥18 years) diagnosed with early or locally advanced uterine cervical cancer will be assessed in the review. We will exclude studies involving exclusively metastatic disease or cases where radiation treatment has not been offered or discussed

Intervention (I): HIV-positive cervical cancer patients in early or locally advanced cervical cancer stages treated with radiation or chemoradiation therapy

Comparator (C): HIV-positive cervical cancer patients in early or locally advanced cervical cancer stages treated with radiation or chemoradiation therapy

Outcomes (O): Primary outcomes: clinical outcomes of cervical cancer treatment with radiation or chemoradiation therapy by HIV status. Secondary outcomes: toxicity profiles

Setting

There will be no restrictions on the type of setting.

Language

We will include all articles published in English.

Search Methods for the Identification of Studies

Search strategy: Studies will be reviewed from the MEDLINE and EMBASE research literature databases. We will also hand-search citation lists of included studies and previous topic-related reviews to identify additional potential studies.  We will run the search strategy from the time of database inception to the current date of extraction, that is, 31st May 2023. A repeat literature search will be conducted before publication of the results to identify new studies.

Study records: The results of the search will be uploaded to Covidence (covidence.org), a web-based software platform that helps streamline systematic reviews. Studies will be selected for inclusion following a three-stage process in Covidence:

Duplicates from the mentioned databases will be filtered out.

Two independent reviewers will screen the titles and abstracts of all the studies. Studies that do not meet the eligibility criteria will be excluded. Conflict or discrepancy will be resolved through mutual discussion.

The full-text articles of all screened studies from the second stage will be retrieved, and final inclusion or exclusion decisions will be made by examining the full-text manuscripts. Two reviewers will then independently select studies that meet the predefined eligibility criteria and record the reason(s) for excluded studies. Any differences in these decisions will be discussed or resolved by a third reviewer. A flow chart of included and excluded studies at various stages of selection will be made following the PRISMA 2009 flow diagram. 

Data extraction: We have designed a data extraction form that will be recreated in Covidence software and made accessible to all authors. The authors will independently perform data screening and extraction. The data to be extracted will include: study title, authors, year of publication, country, study design and methodology, patient demographics, sample size (by HIV status, RT vs chemoRT, treatment doses, CD4 cell counts, viral load [VL] in HIV+), ART regimen, cancer stage, treatment outcomes, toxicities, duration of follow-up, and statistics such as odds ratio, risk ratio, linear and logistic regression, hazard ratio, confidence intervals, and potential confounders. Any discrepancies in the data extraction process will be resolved through discussion and consensus with the expert reviewer.

Quality appraisal: Two reviewers will independently assess the risk of bias for each included study. Observational studies selected for the review will be evaluated using the Newcastle-Ottawa Quality Assessment Scale for Cohort Studies, and randomized controlled trials will be evaluated using the Cochrane Risk of Bias tool.  Discrepancies in the risk of bias assessment will be resolved through mutual discussion among the two reviewers or, where necessary, by consensus of a third reviewer. A copy of the completed checklists will be published with the review results as an additional file.

Data synthesis: Clinical outcomes and toxicities will be compared between the HIV-positive and HIV-negative groups using the risk ratio. We will calculate the pooled OS to assess the association between HIV status and overall survival. A meta-analysis will be done, and the results will be presented as forest plots. Statistical heterogeneity will be tested using the Q-statistic (I2) and chi-squared (χ2) values. An I2 > 75% and χ2 with p < 0.05 will be used to define heterogeneity; I2 values below 40% will be considered unimportant heterogeneity. Where heterogeneity is identified and if the number of studies is adequate, we will conduct subgroup analyses using random effects models by: study design in the case of methodological heterogeneity, type of therapy (radiotherapy or chemotherapy), cervical cancer stage, ART vs no ART, ART regimen (where specified), and study location (developed vs developing countries), where possible. Sensitivity analysis will also be used to identify any specific studies driving heterogeneity.

We will evaluate for outcome reporting bias and compare the fixed-effect estimate with the random-effects estimate to assess the possible presence of small sample bias in the published literature (i.e., where the intervention effect is more beneficial in smaller studies). In the presence of small-sample bias, the random-effects estimate of the intervention is preferable to the fixed-effect estimate. The potential for reporting bias will be further explored using funnel plots if more than 10 studies are available.

Appendix B: R code for risk ratio calculations

# Install required packages if missing

if (!require(meta)) install.packages("meta", dependencies = TRUE)

if (!require(metafor)) install.packages("metafor", dependencies = TRUE)

library(meta)

library(metafor)

# upload dataset

data <- read.csv("dataset.csv")

# RR

meta_analysis <- metabin(

  event.e = Events_HIV,

  n.e = N_HIV,

  event.c = Events_noHIV,

  n.c = N_noHIV,

  studlab = Study,

  data = data,

  sm = "RR",            

  method.tau = "DL",       # DerSimonian-Laird estimator

  comb.fixed = FALSE,      # Random-effects model

  comb.random = TRUE,

  hakn = TRUE,                  # Hartung-Knapp adjustment for better CI

  prediction = TRUE,

  byvar = data$Subgroup,              # optional subgroup variable

  title = "Meta-analysis of dataset"

)

# View summary results

print(meta_analysis)

summary(meta_analysis)

# Forest plot

forest(

  meta_analysis,

  sortvar = TE,               # Sort by effect size

  comb.random = TRUE,

  leftcols = c("studlab", "event.e", "n.e", "event.c", "n.c"),

  leftlabs = c("Study", "Events", "Total", "Events", "Total"),

  rightlabs = c("RR [95% CI]"),

  label.e = "HIV+",

  label.c = "HIV-",

  print.tau2 = TRUE,

  col.diamond = "firebrick",

  col.diamond.lines = "black",

  col.square = "navy",

  xlab = "Risk Ratio (log scale)",

  smlab = "HIV+ vs HIV-",

  overall = TRUE,

  overall.hetstat = TRUE,

  test.overall = TRUE,

  test.subgroup = TRUE

)

# Subgroup analysis

if ("Subgroup" %in% names(data)) {

  print("Subgroup analysis results:")

  print(meta_analysis$byvar)

  print(meta_analysis$Q.b.random)  # Test for subgroup differences

}

#Funnel plot and publication bias test

funnel(

  meta_analysis,

  xlab = "Log Risk Ratio",

  ylab = "Standard Error",

  studlab = TRUE,

  pch = 21,

  bg = "lightblue"

)

# Egger’s test for small-study effects

metabias(meta_analysis, method.bias = "linreg")

# sensitivity analysis (leave-one-out)

leave1out_results <- metainf(meta_analysis)

forest(leave1out_results, pooled.totals = FALSE,

       main = "Leave-one-out sensitivity analysis")
